# Proteomics uncover EPHA2 as a potential novel therapeutic target in colorectal cancer cell lines with acquired cetuximab resistance

**DOI:** 10.1007/s00432-022-04416-0

**Published:** 2022-11-19

**Authors:** Lucien Torlot, Anna Jarzab, Johanna Albert, Ágnes Pók-Udvari, Arndt Stahler, Julian Walter Holch, Marco Gerlinger, Volker Heinemann, Frederick Klauschen, Thomas Kirchner, Jörg Kumbrink, Bernhard Küster, Andreas Jung

**Affiliations:** 1 Institute of Pathology, Ludwig-Maximilians-University (LMU), Munich, Germany; 2 German Cancer Consortium (DKTK), Heidelberg, Munich Site, Germany; 3 Chair or Proteomics and Bioanalytics, Technical University of Munich, Freising, Germany; 4 Department of Hematology, Oncology, and Cancer Immunology, Charité - Universitätsmedizin Berlin, Corporate member of Freie Universität Berlin and Humboldt-Universität zu Berlin, Berlin, Germany; 5 German Cancer Consortium (DKTK), Heidelberg, Berlin, Germany; 6 Department of Medicine III, LMU Hospital, Munich, Germany; 7 Comprehensive Cancer Center Munich (CCCM), LMU Hospital, Munich, Germany; 8 Translational Oncogenomics Lab, The Institute of Cancer Research, London, UK; 9 Barts Cancer Institute, Queen Mary University of London, London, UK; 10 Gastrointestinal Cancer Unit, St Bartholomew’s Hospital, London, UK

**Keywords:** Colorectal cancer, EPHA2, Cetuximab resistance, Molecular oncology, Proteomics

## Abstract

**Background:**

In metastatic colorectal cancer (mCRC), acquired resistance against anti-EGFR targeted monoclonal antibodies, such as cetuximab (CET), was shown to be frequently caused by activating alterations in the *RAS* genes *KRAS* or *NRAS*. To this day, no efficient follow-up treatment option has emerged to treat mCRC in such a setting of resistance.

**Methods:**

To uncover potential targets for second-line targeted therapies, we used mass-spectrometric proteomics to shed light on kinome reprogramming in an established cellular model of acquired, *KRAS*-associated CET resistance.

**Results:**

This CET resistance was reflected by significant changes in the kinome, most of them individual to each cell line. Interestingly, all investigated resistant cell lines displayed upregulation of the Ephrin type-A receptor 2 (EPHA2), a well-known driver of traits of progression. Expectedly resistant cell lines displayed increased migration (*p* < 0.01) that was significantly reduced by targeting the EPHA2 signalling axis using RNA interference (RNAi) (*p* < 0.001), ephrin-A1 stimulation (*p* < 0.001), dasatinib (*p* < 0.01), or anti-EPHA2 antibody treatment (*p* < 0.001), identifying it as an actionable target in mCRC with acquired CET resistance.

**Conclusion:**

These results highlight EPHA2 and its role in mCRC with *KRAS*-gene mutated acquired CET resistance and support its use as a potential actionable target for the development of future precision medicine therapies.

**Supplementary Information:**

The online version contains supplementary material available at 10.1007/s00432-022-04416-0.

## Introduction

Colorectal cancer (CRC) is the fourth-deadliest cancer-related cause of death in men and women worldwide causing approximately 900,000 deaths yearly (Ferlay et al. 2020). Typically primary cause of death is linked to disease progression under treatment and metastatic dissemination with incurring multi-organ failure. Disease progression and treatment resistance have been linked to a multitude of factors, ranging from genomic alterations (e.g., *RAS* genes, involved in the MAPK pathway), gene expression changes (e.g., overexpression of the *TYMS* gene under 5-FU therapy), or changes in DNA methylation (Misale et al. 2012; Jeught et al. 2022; Lu et al. 2016). Resistance against targeted anti-epithelial growth factor receptor (EGFR) therapies has been linked to tyrosine kinase overexpression (HER2 but also recently EPHA2), genetic resistance drivers (mainly the *RAS* genes *KRAS* and *NRAS*, but also *BRAF* or *NF1*), and certain transcriptomic Consensus Molecular Subtypes (CMS) (Martini et al. 2019, 2020; Hahn et al. 2017; Efstathiou, et al. 2022). Alterations in the *RAS* genes have long been known to hold a central role in the oncogenesis of CRC (Fearon and Vogelstein 1990). Primary *RAS* alterations predict poor treatment response to cetuximab (CET), an approved EGFR specific antibody which is used for the treatment of left-sided *RAS*- and *BRAF* wild-type metastatic CRC (mCRC), and were also shown to contribute to acquired (secondary) CET resistance (Misale et al. 2012; Cutsem et al. 2009, 2011; Khambata-Ford et al. 2007; Roock et al. 2010). Oncogenic *KRAS* alterations (mostly codon 12 or 13 mutations) induce over-activation of the MAPK pathway and by crosstalk also other pathways, including the PIK3–AKT–mTOR pathway beside others (Pylayeva-Gupta et al. 2011; Cox and Der 2010). Due to this complexity, it is still unclear how activation of KRAS confers CET resistance and drives disease progression on a molecular level and if this understanding might result in the identification of treatment strategies to overcome disease progression in mCRC.

To address this question, we applied mass spectrometry-based proteomics and studied underlying CET resistance in CRC cell lines displaying CET resistance induced by activating *KRAS* alterations with a special focus on kinome reprogramming. In this study, we found that CET-resistant tumour cell lines (Lim1215 and DiFi) commonly displayed EPHA2 overexpression, a targetable driver of cellular motility and migration.

## Materials and methods

### Cell culture and genetic analyses of cell lines

**Fig. 1:**
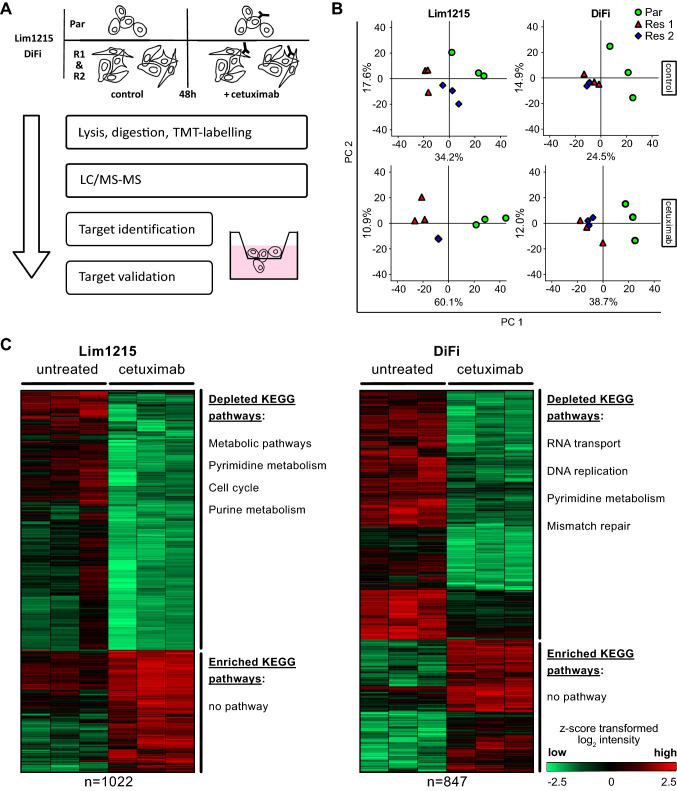
LC–MS/MS-based proteomic characterisation of CRC cell lines and cetuximab (CET) mode of action. **A** Experimental design of liquid chromatography–tandem mass spectrometry (LC/MS–MS)-based characterisation of CET resistance in Lim1215 & DiFi cell lines, as described in the Materials and methods section. **B** Principal component analysis shows solid clustering of biological replicates together as well as clustering of both resistant compared to parental cell lines for both Lim1215 and DiFi. **C** Unsupervised clustering and KEGG annotation enrichment analysis of significantly changing proteins upon CET treatment in parental Lim1215 and DiFi cells. Log_2_ expression intensities were z-score transformed (Benjamini–Hochberg-FDR = 0.05; S0 = 0.1). KEGG annotation enrichment analysis using the STRING database yielded several pathways depleted by CET. Enrichment analysis did not yield any pathways enriched by CET treatment

Lim1215, Lim1215-R1, Lim1215-R2, DiFi, DiFi-R1, and DiFi-R2 cell lines have been described previously (Misale et al. 2012), and were kindly provided by Dr. Alberto Bardelli (Candiolo Cancer Institute, Italy). Lim1215 & Lim1215-R cell lines were grown in RPMI-1640 medium (Biochrom, Berlin, Germany) supplemented with 5% (v/v) FBS, 1% (v/v) penicillin/streptomycin (Biochrom), and 1 µg/ml recombinant insulin (Thermo Fisher Scientific, Waltham, MA, USA). DiFi & DiFi-R cell lines were grown in DMEM/Ham F-12 medium (Biochrom) supplemented with 10% (v/v) FBS and 1% (v/v) penicillin/streptomycin. Genomic DNA from cell lines was prepared using QIAquick DNA extraction kits (Qiagen, Hilden, Germany) according to the manufacturer’s instructions. Cell line identity was confirmed by Short Tandem Repeat (STR) analyses on an ABI 3130 genetic analyzer (Applied Biosystems, Thermo Fisher Scientific, Waltham, MA, USA) employing a commonly used set of 9 STR-markers (Dirks and Drexler 2013). Additionally, all cell lines were submitted to NGS analysis using the OncoMine™ Focus Assay on an IonTorrent™ PGM (personal genome machine; Thermo Fisher Scientific). Lim1215-R and DiFi-R were cultured continuously in the presence of 25 µg/ml cetuximab. All cell lines were routinely tested for *Mycoplasma* contamination utilising PCR Mycoplasma kits (AppliChem, Darmstadt, Germany). Experiments were performed in biological replicates using cells at different passages (5–25). Cetuximab was acquired from the LMU Hospital pharmacy and dasatinib purchased (MedChemExpress, Monmouth Junction, NJ, USA). To uncover kinase reprogramming in resistant cells parental and resistant cells were seeded at 10^6^) cells in 75 cm^2^ flasks and grown in the presence/ absence of 5 µg/mL cetuximab for 48 h before being subjected to cell lysis (Fig. 1A).

### Cell lysis

For LC–MS/MS, proteomic analysis cells were washed twice with ice-cold PBS and lysed in 500 µl of 8 M urea buffer in 80 mM Tris–HCl, pH 7.6, supplemented with protease (Hoffmann-La Roche, Basel, Switzerland) and phosphatase inhibitors (Sigma-Aldrich, St. Louis, MO, USA). Lysates were cleared by centrifugation at 10^5^ ×*g* for 30 min at 4 °C. Protein concentration was determined by Bradford assay (Coomasie Protein Assay Reagent, Thermo Fisher Scientific) (Bradford 1976). For Western blot analysis, cells were lysed in RIPA buffer (Cell Signaling Technology, Danvers, MA, USA) supplemented with protease and phosphatase inhibitors (Hoffmann-La-Roche).

### Proteolysis, TMT-labelling, and peptide fractionation

100 µg protein lysates from each sample were reduced with 10 mM DTT for 45 min at 37 °C and alkylated with 55 mM chloro-acetamide for 30 min at room temperature in the dark. Samples were diluted with 5 volumes of 40 mM Tris–HCl, pH 7.6 and hydrolyzed with trypsin (Promega, Mannheim, Germany) in a 1:50 (w/w) enzyme-to-substrate ratio during overnight incubation at 37 °C in a thermoshaker at 700 rpm. Samples were acidified with formic acid (FA) to a concentration of 0.5% (v/v). Samples were desalted using self-packed stage-tips [10 discs, Ø 1.5 mm, C18 material, 3 M Empore™ Octadecyl C18, Saint Paul, MN, USA; wash solvent: 0.1% formamide (FA); elution solvent: 60% acetonitrile (ACN) in 0.1% FA]. 100 µg of protein hydrolysate were dissolved in 50 mM HEPES, pH 8.5 and mixed for 10 min at 20 °C. Tandem Mass Tag (TMT) reagents (Thermo Fisher Scientific) were added to each protein hydrolysate to a final concentration of 11.6 mM and incubated at 400 rpm on a thermomixer for 1 h at 20 °C. Reactions were stopped with 0.4% hydroxylamine (v/v). Labelled peptide solutions were pooled and desalted on Sep-Pak tC18 RP extraction cartridges (Waters Corp., Finglas, Ireland; wash solvent: 0.1% FA; elution solvent: 60% ACN in 0.1% FA). TMT-labelled samples were fractionated using a Dionex Ultimate 3000 HPLC System (Dionex Corporation, Idstein, Germany) and collected in 32 fractions.

### LC–MS/MS analysis

1 µg of each fraction was injected into an Ultimate 3000 SD HPLC (Thermo Fisher Scientific) coupled to a Q-Exactive HF (Thermo Fisher Scientific) which briefly was operated in data-dependent acquisition and positive-ion mode, automatically switching between MS1 and MS2. For more details, refer to supplementary materials and methods.

### Database searching and data analysis

Peptide and protein identification and quantification were performed using MaxQuant (v1.5.5.1) with embedded Andromeda search engine (Cox et al. 2011). Spectra were searched against the UniProt databases (human, 48,556 entries, download: 19.07.2017). For statistical analysis, the results were imported into Perseus (v.1.5.4.1) (Tyanova et al. 2016). Samples from both resistant cell lines were grouped and compared to their parental counterpart (Lim1215 vs Lim1215-R and DiFi vs DiFi-R). A permutation-based false discovery rate (FDR) adjusted two-sided Student’s *t*-test was used to assess statistical significance (FDR < 0.05, S0 of 0.1).

### Transfection of small interfering RNA

*EPHA2* knock-down was performed using *EPHA2* specific small interfering RNA (siRNA, Qiagen, sequences: supplementary materials) or scrambled siRNA control (Thermo Fisher Scientific). siRNA was transfected at 10 µM using Lipofectamine RNAiMAX (Thermo Fisher Scientific) following the manufacturer’s instructions. Briefly, cells were seeded 24 h before transfection at a density of 8 × 10^5^ cells/ 25 cm^2^ flask. siRNA and Lipofectamine RNAiMAX were diluted in serum-free medium (OPTI-MEM, Biochrom GmbH, Berlin, Germany) and applied with fresh medium to cells. Controls were transfected with scrambled siRNA (Thermo Fisher Scientific). Cells were used for subsequent analyses 24 h after transfection.

### Ephrin-A1-Fc treatment

Cells were stimulated using recombinant ephrin-A1-Fc chimera (ephrin-A1-Fc) or IgG1-Fc control (Fc) (R&D Systems, Minneapolis, MN, USA). Cells were seeded 24 h prior to treatment. Fresh medium was supplemented with 0.1 µg/ml ephrin-A1-Fc or Fc. Assays were performed 24 h after cell stimulation.

### EPHA2-antibody treatment

Cells were seeded 24 h prior to treatment with polyclonal goat anti-EPHA2 (α-EPHA2, AF3035, R&D Systems, directed against EPHA2's extracellular domain) or anti-GFP (α-GFP AF4240, R&D Systems) antibodies at 5 µg/ml. Cells were used in assays 24 h after stimulation.

### Migration assay

Pre-treated cells (EPHA2 knock-down, ephrin-A1-Fc antibody treatment) were seeded after 24 h in Transwell® inserts (Corning, NY, USA) at 1 × 10^5^ cells/insert in low FBS medium (0.25% (v/v) FBS in RPMI or 0.5% in DMEM/Ham F-12). High FBS medium (10% (v/v) FBS in RPMI or 20% (v/v) in DMEM/Ham F-12) was used as a chemoattractant. High FBS medium was supplemented with ephrin-A1-Fc/Fc (0.1 µg/ml) for Ephrin A1-stimulated cells or α-EPHA2/α-GFP (5 µg/ml) for antibody-treated cells. Cells were fixed after 72 h using 100% methanol and stained with crystal violet blue. Non-migrated cells were removed from the insert using cotton swabs. Three representative pictures were taken from each membrane and cell density was assessed using ImageJ (1.49v) (Schneider et al. 2012). Migration data were analysed using an unpaired two-sided Student’s *t*-test using GraphPad Prism (v.8.2.1).

### Western blotting

Cell lysates were boiled in Lämmli buffer (Bio-Rad Laboratories Inc., Hercules, CA, USA) for 10 min at 95 °C, separated on 10% SDS-PAGE gels and transferred onto poly-vinylidene difluoride (PVDF) membranes (Bio-Rad Laboratories Inc.). Membranes were blocked using 5% (w/v) non-fatty milk powder in TBS-T [0.1% (v/v) tween20] for 1 h and incubated with primary antibodies EPHA2 (1C11A12) or α-tubulin (TU-01) (Invitrogen, Carlsbad, CA, USA) overnight at 4 °C. Secondary antibodies (Acris Antibodies GmbH, Hiddenhausen, Germany) were incubated for 1 h, membranes were washed and submerged in chemoluminescent reagent (Millipore, Burlington, MA, USA). Finally, proteins were visualised and quantified using a LI-COR Odyssey FC Scanner in combination with Image Studio (v.5.2, LI-COR Biosciences, Lincoln, NE, USA).

### RNA extraction, RT-PCR, and qPCR

RNA was isolated using RNeasy Mini Kits (Qiagen). RT-PCR was performed with 1 µg RNA using RevertAid Reverse Transcriptase (Thermo Fisher Scientific) and random hexamer primers (Thermo Fisher Scientific) following the respective manufacturer’s instructions. Real-time quantitative PCR was performed using the Universal ProbeLibrary system (UPL, Hoffmann-La Roche) with Probe Master reagents (Hoffman-La Roche) and gene specific primers (supplementary material and methods) according to the manufacturer’s instructions. Gene expression levels were calculated applying the ΔΔCt method, thereby normalising to GAPDH expression levels.

### Cell viability assay

Drug sensitivity was measured by treating each cell line with increasing concentrations of CET or dasatinib. In short, cells were seeded in quadruplicates at 3–4 × 10^3^ cells per well in 96-well plates. Drugs or vehicle control was added 24 h later at increasing concentrations. Cells were treated for 48 h (CET) and 72 h (dasatinib) before adding 10 µl alamarBlue™ reagent (Thermo Fisher Scientific) to each well. Fluorescence was measured after 4 h using a Varioskan plate reader (Thermo Fisher Scientific). Cell viability and half maximal inhibitory concentrations (IC_50_) were assessed using GraphPad Prism (v.8.2.1).

### Clinical study

For translating experimental data into a clinical context, results from the Prospect-C study were included (Martini et al. 2019). This study was performed in accordance with the protocol and in compliance with the Declaration of Helsinki and was approved by UK Research Ethics Committee 127LO/914. All patients provided written informed consent before trial entry.

## Results

### LC–MS/MS-based proteomics characterise cetuximab-resistant cell lines

To investigate mechanisms of *KRAS*-associated cetuximab (CET) resistance, we used an established cell culture model of acquired CET resistance consisting of isogenic CET sensitive and resistant Lim1215 and DiFi colorectal cancer (CRC) cell lines (Misale et al. 2012). Resistance was conferred through activating alterations of *KRAS* (point mutations in Lim1215-R or amplifications in DiFi-R-resistant cell lines) found in each two independently generated clones of the two cell lines. In a first step, we reviewed the characteristics of parental (Par, sensitive) and resistant Lim1215 (Lim1215 R1 & R2) and DiFi (DiFi R1 & R2) cell lines. Short tandem repeat (STR) profiles (data not shown), genetic alterations (Supplementary table 1), and CET sensitivity (Supplementary fig. S1A & S1B) confirmed both identity and behaviour of all cell lines as expected. We next started the proteomic analysis to identify molecular effects of KRAS signalling on kinase reprogramming in Lim1215-R and DiFi-R cell lines. Protein lysates of CET-treated (48 h) or -untreated Lim1215 and DiFi clones were subjected to liquid chromatography–tandem mass spectrometry (LC–MS/MS) (Fig. 1A). Our setup collected quantitative data for as many as 7000 different proteins and 200 kinases across all samples. First, we assessed the technical quality of the experimental approach by correlating data from individual replicates with one another. The correlation within biological replicates (Pearson's *R* > 0.98) was excellent (Supplementary fig. S2A-S2D). Technical quality was further validated using principal component analyses (PCA), which resulted in a solid clustering of samples by biological replicates (Fig. 1B). Furthermore, in the PCAs, samples from the respective two resistant cell lines clustered more tightly to one another than to samples from their parental cell line. This supported the good quality of the data yielded from individual replicates and pointed to a similar biology despite resistant clones having been generated independently. Taken together, these results validate the proteomic data as being of sufficient high technical quality to be reliably used in subsequent analyses.

### Chemical proteomics confirm cetuximab mode of action

Using the expected CET mode of action as an additional quality parameter, we next compared CET-treated to untreated parental cell lines. Protein expression changes induced by CET were identified using a two-sided false discovery rate (FDR) controlled *t*-test (FDR < 0.05, S0 of 0.1). Unsupervised hierarchical clustering was performed and enrichment analysis of changing proteins using the STRING database (Szklarczyk et al. 2017) in combination with KEGG annotations (Kanehisa and Goto 2000) shed light into biological effects of CET treatment (Fig. 1C). Expectedly, the cell division cycle was significantly diminished in both cell lines when treated with CET (FDR_Lim1215_ = 6.55 × 10^–5^, FDR_DiFi_ = 4.35 × 10^–5^) which was reflected by depletion of key cell cycle proteins, such as MYC, MAPK14, cyclins, and cyclin-dependent kinases (CCND1, CCNA2, CCNH, CDK2) as well as EGFR in DiFi. It is well known that loss of EGFR upon CET treatment occurs by receptor internalisation and degradation (Vincenzi et al. 2010; Okada et al. 2017). Additionally, other pathways were affected by CET treatment. In Lim1215 cells, metabolic- (FDR_Lim1215_ = 3.05 × 10^–10^), AMPK- (FDR _Lim1215_ = 5.35 × 10^–3^), insulin- (FDR _Lim1215_ = 0.0106), and mTOR- (FDR _Lim1215_ = 0.042) signalling pathways were depleted. This was reflected by the reduction of PI3K and AKT1 expression, both involved in mTOR signalling (Scott et al. 1998; Thomas et al. 2002). In DiFi, mismatch repair- (FDR_DiFi_ = 0.0018) and FoxO- (FDR_DiFi_ = 0.0472) signalling pathways were depleted. These data support the biological quality of the proteomic approach by validating the coherence of the generated dataset and known biological effects of CET (Vincenzi et al. 2010; Russo et al. 2022). However, they also showed that specific targeting of the EGFR–RAS–RAF–MAPK axis resulted in a heterogeneous molecular response involving various signalling pathways in the two parental cell lines.

### Cetuximab resistant cells differ from parental cells in their response to treatment

**Fig. 2:**
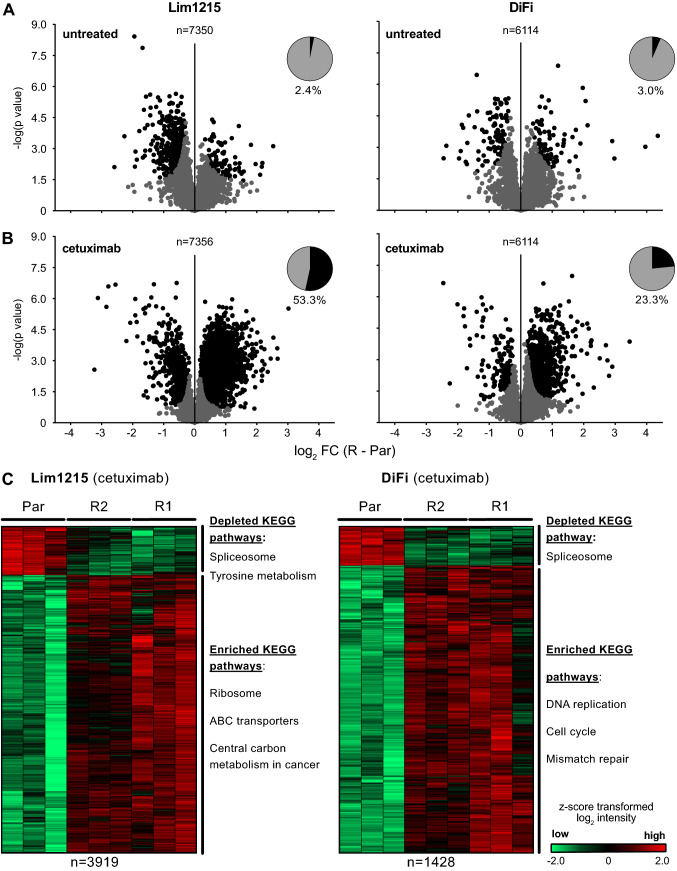
LC–MS/MS-based proteomics identifies expression changes in pathways associated with hallmarks of cancer. **A, B** Volcano plots of protein expression changes in resistant cells compared to parental cells. Expression differences were plotted as log_2_ fold-change (FC) against the significance of the difference (FDR < 0.05; S0 of 0.1; black dots). Both resistant cell lines (R) were jointly compared to their parental counterpart (Par) in the untreated **A** and CET-treated **B** state. **C** Unsupervised clustering and KEGG annotation enrichment analysis of significantly deregulated proteins in CET-treated resistant Lim1215 and DiFi cells. Log_2_ expression intensities of differentially expressed proteins were z-score transformed. KEGG term enrichment analysis using the STRING database shows various pathways associated with hallmarks of cancer enriched in Lim1215-R and DiFi-R cell lines

Having confirmed the technical and biological reliability of our data, we next searched for protein expression changes occurring in resistant cells. In search of overlapping molecular reprogramming protein expression levels in both resistant cell lines were compared to their respective parental counterparts in Lim1215 and DiFi (FDR controlled *t*-test, FDR < 0.05, S0 of 0.1). Protein expression levels differed little when comparing resistant and parental cells in the untreated setting as resistance accounted for significant expression differences in only 2.4% (Lim1215) and 3.0% (DiFi) of all identified proteins, respectively (Fig. 2A). However, when treated with CET, resistant and parental cell lines differed significantly in 53.3% and 23.3% proteins for Lim1215 and DiFi respectively (Fig. 2B). These results show that CET-resistant clones differ from their parental counterpart in their reaction to CET treatment, but show little innate differences in protein expression.

We next sought to uncover changes in signalling pathways in resistance. Enrichment analysis based on the STRING database was therefore applied to Lim1215-R and DiFi-R. In CET-treated Lim1215-R cell lines resistance was accompanied by enrichment of ribosomal proteins (FDR = 8.86 × 10^–8^), ABC transporters (FDR = 5.10 × 10^–3^) and metabolic proteins (FDR = 3.64 × 10^–2^). CET-treated DiFi-R cells displayed enrichment of DNA replication (FDR = 8.50 × 10^–4^), cell cycle (FDR = 6.60 × 10^–3^) and DNA-mismatch repair proteins (FDR = 1.12 × 10^–2^) (Fig. 2C). These analyses showed that resistance in Lim1215 and DiFi cell lines is accompanied by individual proteome reprogramming. Taken together, these results demonstrate that Lim1215-R and DiFi-R differ from their respective parental counterpart mainly in their reaction to CET treatment and display individual molecular signatures associated with resistance.

### EPHA2 is overexpressed in resistant Lim1215 and DiFi cells

Having uncovered individual proteomic reprogramming in resistant cells, we searched for common changes occurring in resistance. We focused on protein kinases, as their expression change is known to be a major mediator of kinome reprogramming (Blume-Jensen and Hunter 2001; Fleuren et al. 2016). First, global kinase expression differences between resistant and parental cell lines were identified using an FDR controlled *t*-test (FDR < 0.05; S0 of 0.1). In our approach, overall kinase expression differences were concordant with expression differences in the total proteome as described above. Untreated resistant cell lines displayed little difference from parental cells in kinase expression (Lim1215: 6.3%; DiFi: 3.7%). Kinase expression differences between resistant and parental cells increased almost tenfold in the treated setting (Lim1215: 47%; DiFi: 35%) (Supplementary fig. S3A & S3B), indicating that kinase expression behaves similarly to total proteome changes in resistant cells.

**Fig. 3:**
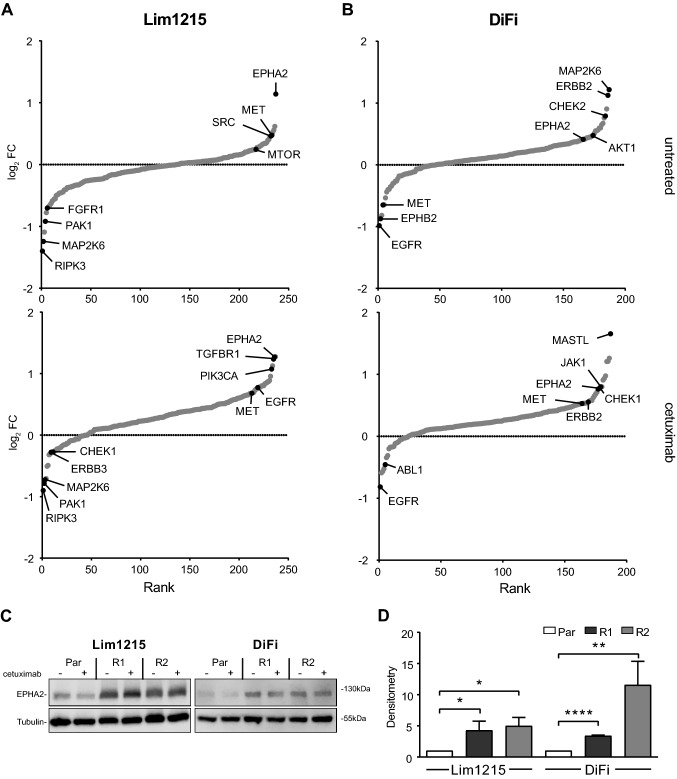
EPHA2 is the most overexpressed kinase in all resistant Lim1215 and DiFi cell lines. **A, B** Rank plot of kinases by log_2_ fold-change (FC) from lowest to highest highlights shows EPHA2 as common overexpressed kinase in Lim1215-R **A** and DiFi-R **B**. **C** Western blot of parental cells (Par) and CET-resistant cells (R1 & R2) confirm EPHA2 overexpression in resistant cells independently of CET treatment. **D** Densitometric data representative of 3 Western blots show EPHA2 overexpression in untreated resistant cell lines (data represent mean ± SEM). Student's *t*-test, *p* < 0.05 in all samples)

To identify potential relevant kinases associated with resistance, kinases were ranked by their log_2_ fold expression change (log_2_ FC) induced in resistance (Fig. 3A, Supplementary table 2). It turned out that kinases with known oncogenic functions (SRC, MET, PIK3CA, AKT1, and EPHA2) were overexpressed in individual resistant cell lines. We found only EPHA2 to be overexpressed in all four resistant cell lines, being the most strongly overexpressed kinase in Lim1215-R (2.4-fold overexpression; *p* = 0.0095) and among the most overexpressed kinases in DiFi-R cell lines (1.7-fold overexpression; *p* = 0.0273). This observation was supported by previous findings that EPHA2 overexpression is a common downstream effect of aberrant RAS signalling in CRC (Dunne et al. 2016; Cuyàs et al. 2017). Overexpression data from mass-spectrometry were confirmed by Western blotting (Fig. 3C). Interestingly, EPHA2 overexpression was independent of CET treatment and was expressed 4.3 (Lim1215-R1), 5.0 (Lim1215-R2), 3.4 (DiFi-R1), and 11.6 (DiFi-R2) times higher than in parental cells (Fig. 3D, p < 0.05). Targeting EPHA2 or a selection of these overexpressed kinases using kinase inhibitors did not significantly affect cell proliferation (data not shown), nor did it restore cetuximab sensitivity. We therefore dismissed them as drivers of cell proliferation in this model of CET resistance and focused on other hallmarks of cancer that drive disease progression, such as cell migration, as EPHA2 is known to regulate cell migration and has been associated with elevated metastatic potential and poor survival in CRC and other malignancies (Xiao et al. 2020).

### EPHA2 is a targetable driver of migration in CET-resistant CRC cell lines

**Fig. 4:**
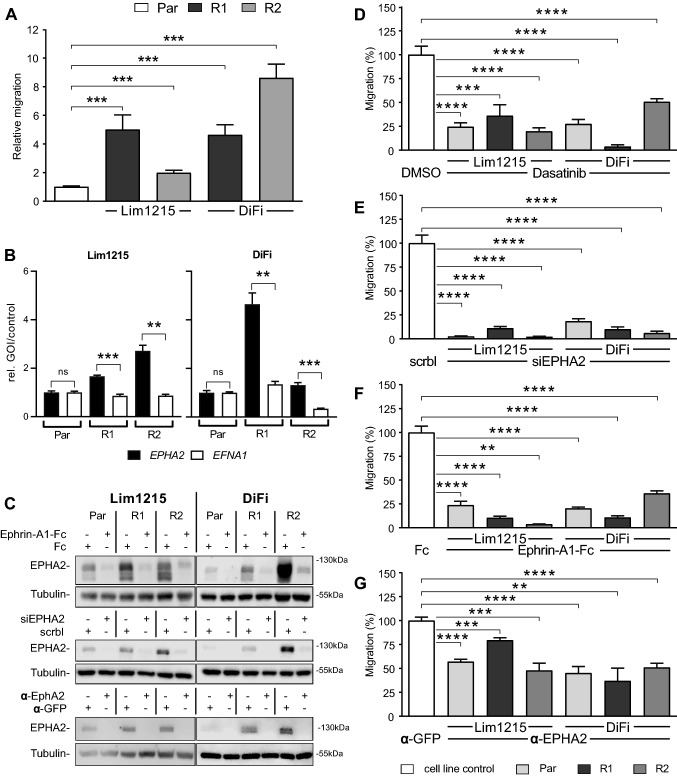
EPHA2 is a specific driver of migration in Lim1215-R and DiFi-R. **A** In both Lim1215 and DiFi, Transwell® assays show migration was significantly higher in resistant cells (R1 & R2) than in parental cells (Par) (4 biological replicates; Student's *t*-test, *p* < 0.001 in all resistant cell lines). **B** RT-qPCR show EPHA2 gene overexpression in resistant cell lines (R1 & R2), while EFNA1 expression remains at parental (Par) baseline level in Lim1215 and DiFi cell lines. **C** Western blots show EPHA2 depletion 24 h after RNAi, ephrin-A1-Fc stimulation, and anti-EPHA2 antibody treatment. Migration rates are significantly decreased by EPHA2 targeting using dasatinib **D,** RNAi **E**, EPHA2 stimulation using recombinant ephrin-A1-Fc **F**, and anti-EPHA2 antibody treatment **G** (representative experiments of 3 biological replicates). Data represent mean ± SEM of three technical replicates. Significance assessed by Student's *t-*test (compared to cell line control,) *p* < 0.01 in all experiments

EPHA2 overexpression was previously linked to increased migration, aggressiveness, and poor survival in CRC (Dunne et al. 2016; Xiao et al. 2020; Robertis et al. 2017). This was also true in the resistant cell lines as they displayed a 5.0- (Lim1215-R1), 2.0- (Lim1215-R2), 4.6- (DiFi-R1), and 8.6-fold (DiFi-R2) higher migration rate in migration assays using Transwell® membranes (Fig. 4A, p < 0.001 for all resistant cell lines).

Active EPHA2 kinase signalling, induced by binding of its ligand Ephrin A1, inhibits migration and MAPK signalling (Dunne et al. 2016; Cuyàs et al. 2017). However, ligand-independent signalling in absence of Ephrin A1 has been known to promote oncogenic signalling and migration, establishing the concept of ligand-receptor imbalance in cancer cells (Miao et al. 2009). Our mass-spectrometric approach did not identify Ephrin A1. However, both *EPHA2* and *EFNA1* (encoding Ephrin A1) were transcriptionally expressed as shown by quantitative real-time PCR (RT-qPCR) showing increased *EPHA2*- and low *EFNA1*-RNA expression in resistant cells (*p* < 0.01, Fig. 4B). These data might support the concept of ligand-independent overexpression of EPHA2 in CET-resistant cells. Furthermore, in previous findings, *EPHA2* high and *EFNA1* low expression in CRC patients was associated with reduced survival (Robertis et al. 2017).

Next, we aimed to target EPHA2 driven migration using dasatinib, a well-known but rather unspecific EPHA2 small molecule inhibitor. Dasatinib reduced migration significantly by 50–90% in resistant cells (*p* < 0.01) at 300 nM, a concentration that did not affect cell viability (Fig. 4D, Supplementary fig. S1C & S1D). As dasatinib targets other kinases as well, we next assessed more specifically whether cell migration was indeed mediated by EPHA2 in the resistant cell lines using RNAi. Silencing EPHA2 expression was highly effective (Fig. 4C and Supplementary fig. S4A & S4B) and reduced migration rates by more than 80% in all resistant cell lines (*p* < 0.001, Fig. 4E). In a third approach, we stimulated EPHA2 using recombinant Ephrin-A1-Fc, as ligand-mediated EPHA2 activation was reported to reduce both EPHA2 mediated migration and adhesion and cause receptor internalisation and degradation (Miao et al. 2000). This was also achieved in all Lim1215-R and DiFi-R cell lines, where Ephrin-A1 stimulation depleted EPHA2 levels (Fig. 4C) and significantly reduced migration by 60–90% in all cell lines (*p* < 0.001) (Fig. 4F). In a fourth approach, we tested a more specific pharmacological treatment option than dasatinib to block the EPHA2 signalling axis using anti-EPHA2-antibody treatment. This approach showed promising results in the treatment of melanoma, breast cancer and gastric cancer (Sakamoto et al. 2018; Hasegawa et al. 2016). We tested this approach in our CRC cell line model by opting for a commercially available Western blotting approved anti-EPHA2 antibody that binds to the extracellular domain of the tyrosine kinase (Materials & Methods). Antibody treatment depleted EPHA2 levels in resistant cells (Fig. 4C) and significantly decreased migration by 20–50% (*p* < 0.001) at 5 µg/ml (Fig. 4G). As mentioned above, targeting EPHA2 did not affect cell viability nor did it restore CET sensitivity in resistant cell lines, dismissing it as a driver of proliferation. However, these data assign EPHA2 a role as a potent driver of migration in CET-resistant CRC cell lines and thus a potential suitable second-line therapeutic option to target disease progression in CET-resistant CRC cell lines.

### EPHA2 may be overexpressed in CRC patients with acquired CET resistance

EPHA2 has recently been identified as a clinically relevant biomarker in CRC patients as a poor prognostic marker in UICC stage II/III CRC patients owing to its ability to promote migration and invasion (Dunne et al. 2016; Robertis et al. 2017; Cioce and Fazio 2021, Figure 1). Moreover, EPHA2 overexpression correlated with disease progression and worse outcome under FOLFIRI plus CET combinational treatment (Martini et al. 2019), thereby attributing EPHA2 a role in (primary) resistance in first-line treatment in mCRC patients. We searched to translate our findings in the clinical setting by investigating the role of EPHA2 in acquired (secondary) CET resistance.

**Fig. 5:**
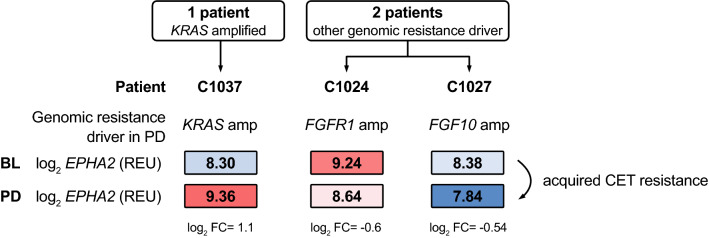
*EPHA2* may be overexpressed in CET-resistant, *KRAS*-altered mCRC patients. Tumour transcriptomic and genomic characteristics of mCRC patients recruited in the Prospect-C trial with acquired CET resistance. *EPHA2* expression level in log_2_ REU (relative expression units). *Amp* gene amplification. *BL* baseline biopsy. *PD* progressive disease biopsy

To translate our experimental results into a clinical setting, we assessed EPHA2 in the context of acquired KRAS mediated CET resistance, searching for transcriptomic data from mCRC patients treated with anti-EGFR therapies that developed secondary resistance. Unfortunately, only very few studies are available in which paired tissue from both pre-treatment with anti-EGFR targeted therapies and after gain of resistance were investigated. We searched for patients within the Prospect-C trial treated with single-agent CET that developed *KRAS* alterations (activating mutation or amplification—*KRAS*mt) as a resistance driver, mirroring our cell culture model of acquired CET resistance. Genomic resistance drivers in the RAS/RAF pathway were identified by tissue biopsy sequencing in 5 of the 14 patients (36%) that had shown prolonged benefit of CET therapy before developing progressive disease (PD) (C1005, C1024, C1025, C1027, C1037) (Suppl. fig. S5) (Woolston et al. 2019). Gene expression data were available for only three of these patients with acquired CET resistance (C1024, C1027, C1037). Comparison of transcriptomic data from baseline biopsies (BL) and progressive disease biopsies (PD) showed a strong *EPHA2* overexpression (log_2_ FC(PD-BL) = 1.06) in the C1037PD sample, which had developed a novel *KRAS* amplification in the PD (similarly to DiFi-R cell lines) (Fig. 5). Samples C1024PD and C1027PD1 displaying other genomic alterations than *KRAS* (FGFR1 amplification and FGF10 amplification respectively) expectedly displayed no EPHA2 overexpression in the PD (log_2_ FC = -0.60 and log_2_ FC = -0.54 respectively) (Fig. 5). This may mirror our cell culture model of *EPHA2* overexpression in *KRAS* amplified or mutated, acquired CET resistance, although despite the perfect match, it could not reach significance due to the small sample size (Fisher’s exact test, *p* = 0.133).

These data indicate that *EPHA2* may be overexpressed in the context of novel *KRAS* alterations in mCRC patients with acquired CET resistance, mirroring the results of our cell-based model of CET resistance. However, considering the small group size, a prospective study involving a larger cohort undergoing CET treatment is inevitably required to reinforce these findings.

## Discussion

In this cell line-based study, KRAS-associated secondary CET resistance was accompanied by considerable reprogramming of both proteome and kinome. Individual resistant cell lines displayed individual or non-functional proteomic changes except for EPHA2, which was overexpressed in all four independently generated resistant cell lines harbouring activating *KRAS* alterations. RAS alterations and EPHA2 might be linked as RAS signalling was shown to induce EPHA2 overexpression via MAPK and RalGDS pathways beside others (Dunne et al. 2016; Cuyàs et al. 2017). This supports our results and the concept that EPHA2 overexpression might be a by-product of overactive RAS signalling in CET-resistant cell lines.

EPHA2 is one of the most prominent EPH receptor family members in cancer, as its overexpression has been linked to treatment resistance, metastatic potential and disease progression in several cancer entities and has been shown to affect both cell proliferation and cell migration (Martini et al. 2019; Xiao et al. 2020; Miao et al. 2009; Zhuang et al. 2010; Koch et al. 2015). In CRC, EPHA2 was found to be a poor prognostic marker in UICC stage II/III disease due to its ability to promote migration and invasion (Dunne et al. 2016). Moreover, EPHA2 correlated to disease progression and worse outcome under first-line FOLFIRI plus CET combination therapy (Martini et al. 2019), attributing EPHA2 a role in primary treatment resistance in mCRC patients. The pro-tumourigenic effects has been linked to ligand-independent EPHA2 signalling, which sustains migration and invasion (Miao et al. 2000, 2001). In our resistant cell lines, *EPHA2* was strongly overexpressed, while *EFNA1* gene expression levels remained at baseline level, mirroring the previous findings of *EPHA2* high and *EFNA1* low expression pattern, associated with reduced survival in CRC patients (Robertis et al. 2017). Our findings support the idea of ligand–receptor imbalance in this cell line model, driving ligand-independent migration, and as expected, all Lim1215-R and DiFi-R cell lines displayed significantly higher migration rates than their parental counterparts (Miao et al. 2009, 2000; Boyd et al. 2014). This increased migration in resistant cells could be successfully inhibited by targeting EPHA2 signalling using dasatinib or by rectifying EPHA2 receptor–ligand imbalance through depletion of EPHA2 using RNAi or antibody treatment, as well as by stimulation with recombinant ephrin-A1. Dasatinib while targeting several other kinases (including BCR-ABL, cKIT, PDGFR, and SRC family kinases) has the advantage of being an FDA and EMA approved drug for the treatment of chronic myeloid leukaemia (CML) with a good oral bioavailability. It was also shown to effectively reduce EPHA2 phosphorylation and activity (Xiao et al. 2020). However, more specific anti-EPHA2 therapeutic options, such as ALW-II-41–27, candidate 4a, and GLPG1790, having emerged in recent years, displaying good in vitro and in vivo efficacy against EPHA2 phosphorylation and affecting epithelial–mesenchymal transition (EMT) and migration in CRC, non-small cell lung cancer (NSCLC), and glioblastoma (Amato et al. 2014; Colapietro et al. 2022; Heinzlmeir et al. 2017).

Previous reports of EPHA2-dependant cell proliferation in the context of CET resistance could not be confirmed in this cell line model. Targeting EPHA2 was unable to restore sensitivity to CET in viability experiments, ruling it out as a resistance driver of proliferation (the driver of proliferation being known to be aberrant RAS signalling in Lim1215-R and DiFi-R cell lines), but rather a targetable driver of migration in resistant cells.

In mCRC patients with acquired CET resistance from the Prospect-C trial, *EPHA2* overexpression was found in one patient displaying a secondary *KRAS* amplification, but not in other patients with other genomic drivers of CET resistance. The progressive disease sample (PD) from patient C1037 may therefore mirror our cell line model and suggests a possible clinical translation of our findings. mCRC patients with KRAS-associated, acquired CET resistance may therefore benefit from second-line anti-EPHA2-targeted therapies, which we showed to be effective in our cell line model. These findings can be interpreted at most as a trend due to the low number of patients.

Taken together, we present evidence that the EPHA2-signalling axis is activated in *KRAS*-altered CET-resistant CRC cell lines, as well as in a mCRC patient with *KRAS*mt-associated acquired CET resistance. This study, supported by previous findings, supports the rationale of EPHA2 targeted therapies in the context of acquired CET resistance to reduce disease progression through cell migration and metastasis (Martini et al. 2019; Dunne et al. 2016; Xiao et al. 2020; Colapietro et al. 2022). Unquestionably this hypothesis needs to be confirmed in larger patient cohorts.

## Supplementary Information

Below is the link to the electronic supplementary material.

Supplementary file1 (PDF 464 kb)

## Data Availability

Mass-spectrometry proteomics data are deposited at the ProteomeXchange Consortium via the PRIDE partner repository, dataset identifier: PXD022072 (Perez-Riverol et al. 2019).
