# Supplementary material for: Proteomics uncover EPHA2 as a potential novel therapeutic target in colorectal cancer cell lines with acquired cetuximab resistance
Source: J Cancer Res Clin Oncol. 2022 Nov 19;149(2):669–82. doi: 10.1007/s00432-022-04416-0 (PMC9931833; doi:10.1007/s00432-022-04416-0)
Supplement: Supplementary file 1 — Supplementary file1 (PDF 464 kb) [file 432_2022_4416_MOESM1_ESM.pdf]

Proteomics uncover EPHA2 as a potential novel therapeutic target in colorectal cancer cell lines with acquired cetuximab resistance

Journal of Cancer Research and Clinical Oncology, 2021

Lucien Torlot[1,3], Anna Jarzab[2], Johanna Albert[1], Ágnes Pók-Udvari[1], Arndt Stahler[4], Julian Walter Holch[3,4,5], Marco Gerlinger[6], Volker Heinemann[3,4,5], Thomas Kirchner[1,3,5], Jörg Kumbrink[1,3,5], Bernhard Küster[2,3], Andreas Jung[1,3,5,7]

[1] Institute of Pathology, Ludwig-Maximilians-University (LMU), Munich, Germany

[2] Chair or Proteomics and Bioanalytics, Technical University of Munich, Freising, Germany

[3] German Cancer Consortium (DKTK), Heidelberg, Munich site, Germany.

[4] Department of Medicine III, LMU Hospital, Munich, Germany

[5] Comprehensive Cancer Center, LMU Hospital, Munich, Germany

[6] Translational Oncogenomics Lab, The Institute of Cancer Research, London, UK

[7] Corresponding author: andreas.jung@lmu.de

Supplementary information

Supplementary figures:

- Supplementary figure S1: Sensitivity to cetuximab and dasatinib of parental and resistant cell lines.
- Supplementary figure S2: Scatter plots of protein expression levels.
- Supplementary figure S3: LC-MS/MS based proteomics identifies overexpressed kinases in resistant cell lines.
- Supplementary figure S4: Successful EPHA2 knockdown using RNAi.

Supplementary tables

- Supplementary table 1: Next generation sequencing results of Lim1215 and DiFi cell lines.
- Supplementary table 2: Kinase overexpression in resistant Lim1215 and DiFi cell lines.

Supplementary materials and methods

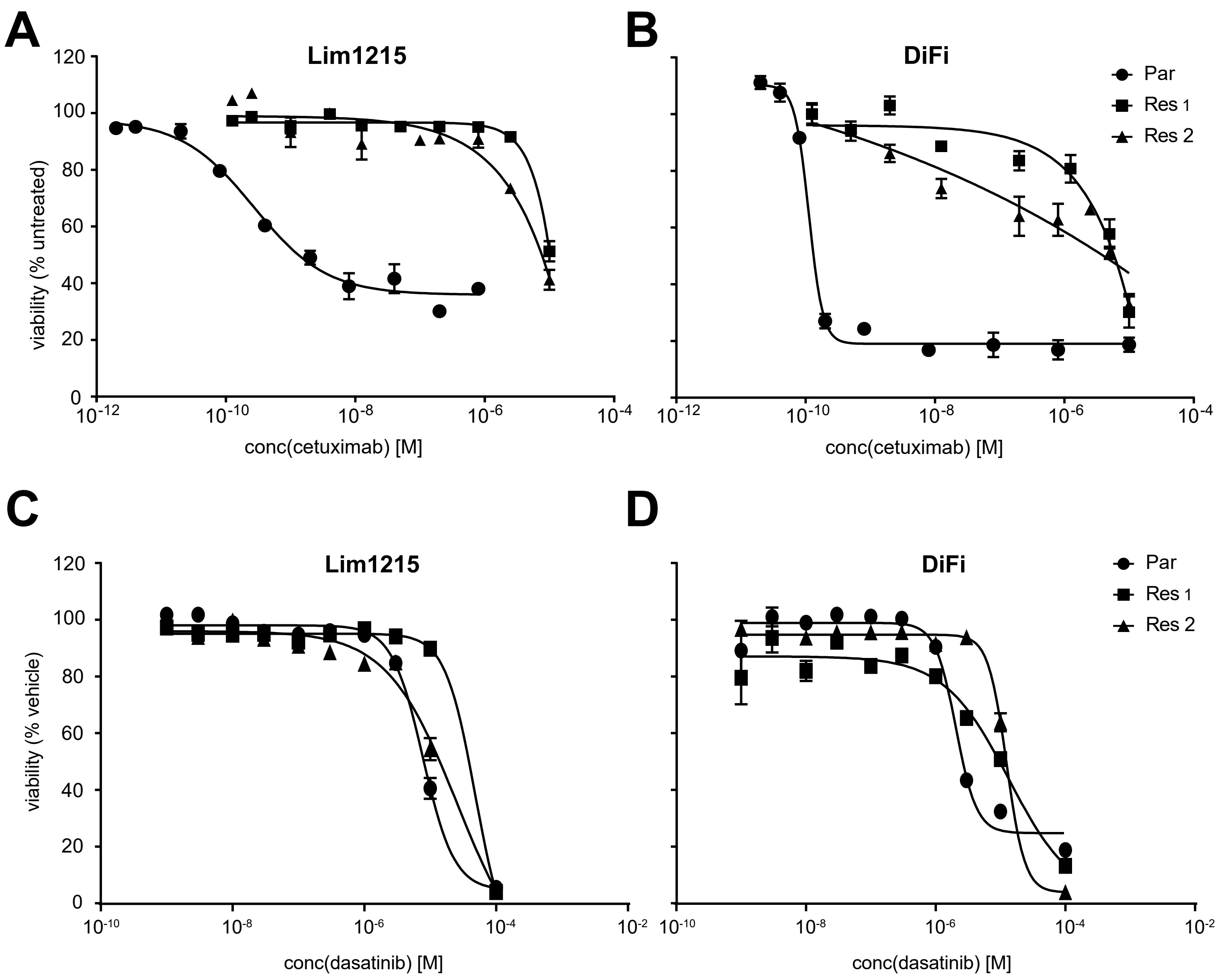

Supplementery figure S1: Sensitivity to cetuximab and dasatinib of parental and resistant cell lines. In Lim1215 and DiFi, parental (Par) and resistant cell lines (R1 & R2) were treated for 48h with increasing doses of cetuximab (**A, B**) or for 72h with increasing doses of dasatinib (**C, D**). Viability was measured using alamarBlue® reagent. Respectively representative of three experiments. Graphed data represents mean relative viability (compared to untreated or vehicle treated cells) ± SEM of 3 technical replicates.

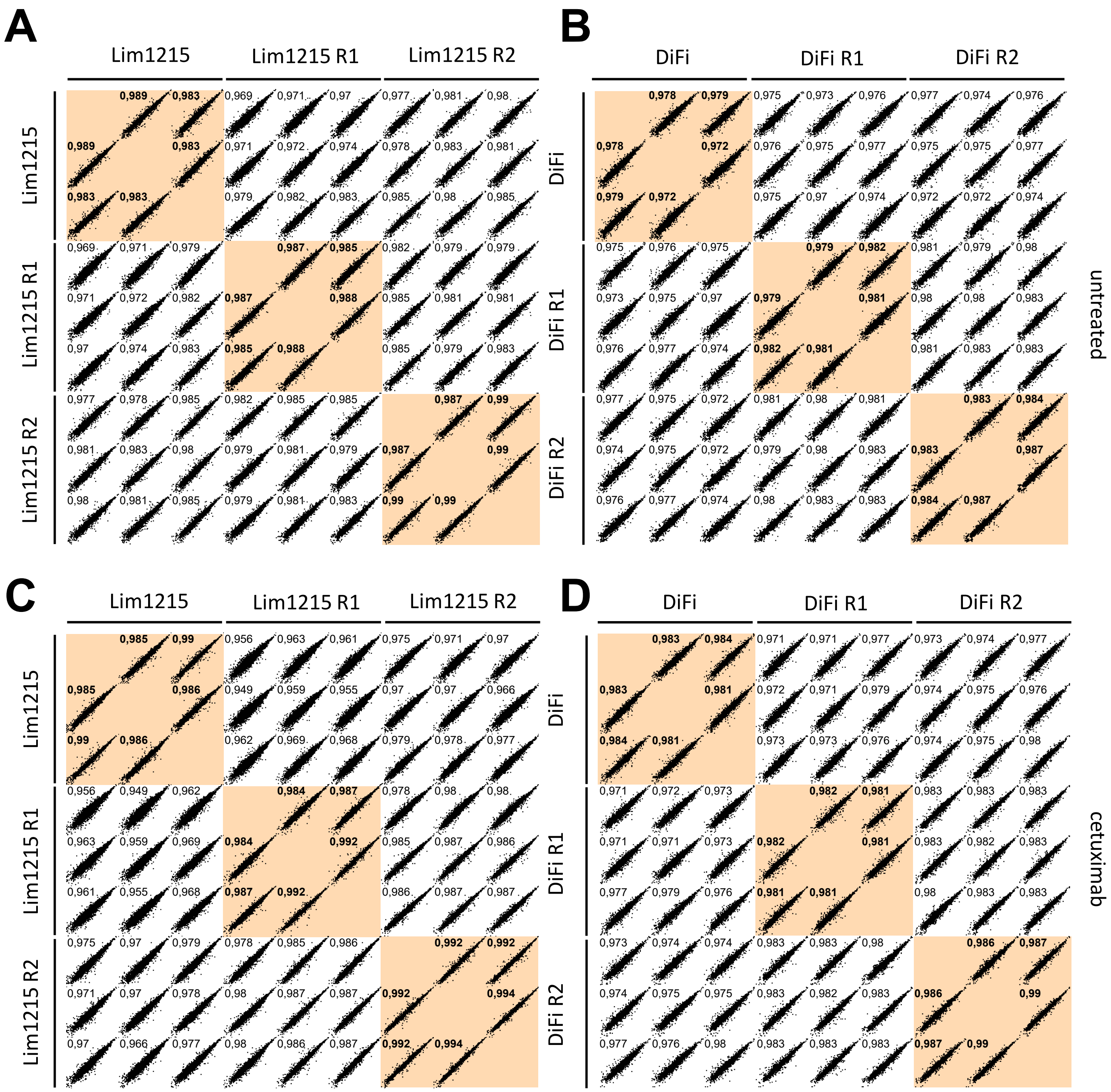

Supplementery figure S2: Scatter plots display the correlation of protein expression data between each of the samples and associated Pearson R. Individual replicates of Lim1215 **(A, C)** and DiFi cell lines **(B, D)** show an excellent correlation of protein expression data (Pearson R > 0.98) according to the experimental setting (untreated **A, B**; cetuximab **C, D**).

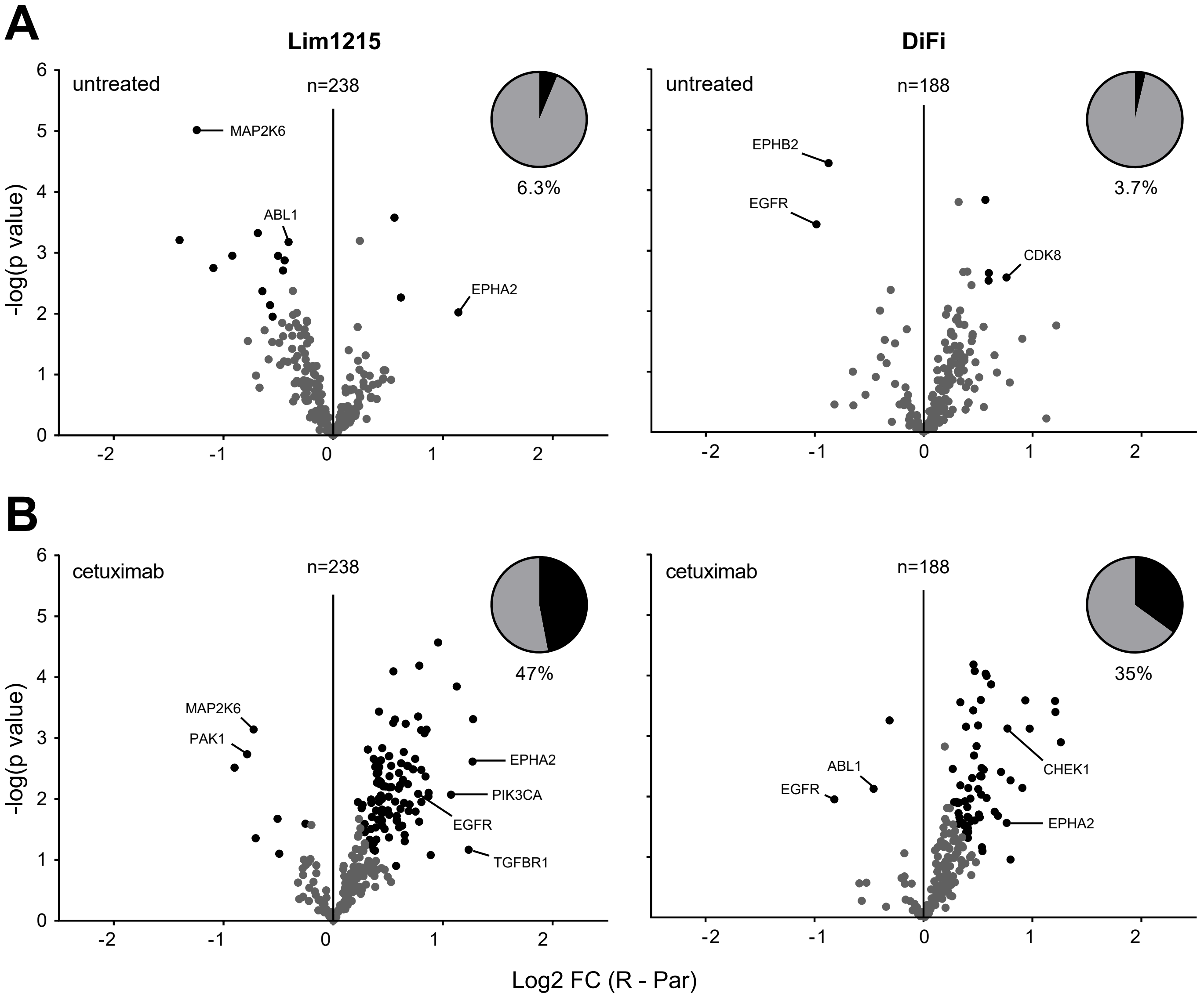

Supplementery figure S3: LC-MS/MS based proteomics identifies overexpressed kinases in resistant cell lines. **(A, B)** Volcano plots of protein kinase expression changes in resistant cells compared to parental cells. Expression differences were plotted as log2 fold-change (FC) against the significance of the difference (FDR<0.05; S0 of 0.1; black dots). Both resistant cell lines (R) were jointly compared to their parental counterpart (Par) in the untreated **(A)** and cetuximab treated **(B)** state.

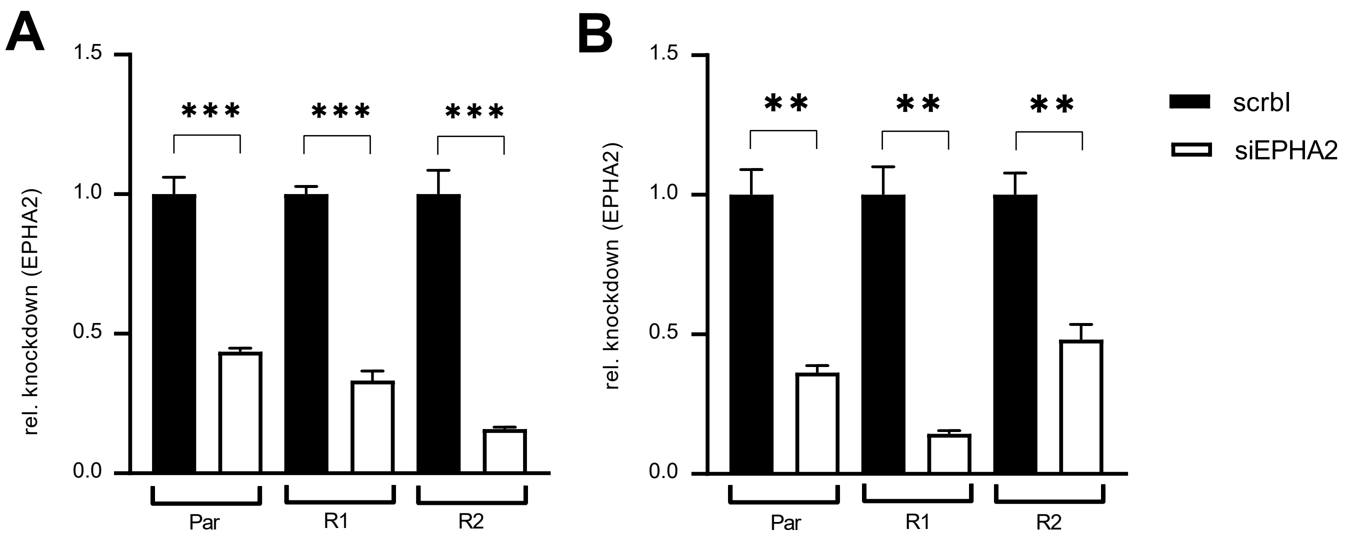

Supplementery figure S4: Successful EPHA2 knockdown using RNAi. RT-qPCR show EPHA2 gene expression knockdown using RNAi is highly successful in all Lim1215 **(A)** and DiFi **(B)** cell lines. Representative of 3 experiments performed in triplicates. Graphs shows mean relative change to parental cell lines ± SEM. Significance assessed by two-sided Student's *t* test.

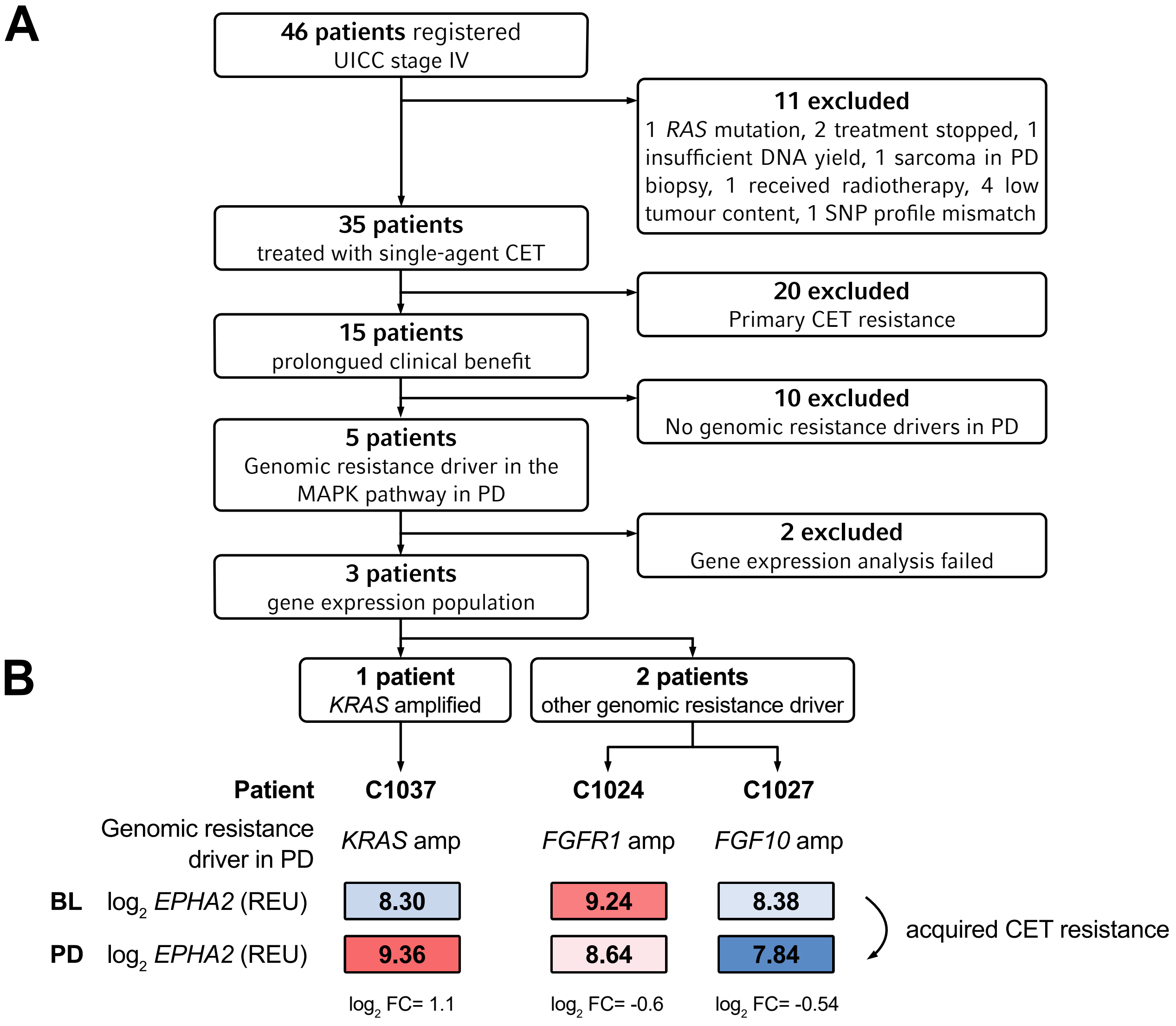

Supplementery figure S5: *EPHA2* may be overexpressed in CET resistant, *KRAS* altered mCRC patients. **(A)** Prospect-C trial CONSORT diagram. **(B)** Tumour transcriptomic and genomic characteristics of mCRC patients with acquired CET resistance. *EPHA2* expression level in $\log_2$ REU (relative expression units). SNP - single nucleotide polymorphism. amp - gene amplification. BL - baseline biopsy. PD - progressive disease biopsy.

cell line	chrom. pos.	gene	cDNA	type	exon	Ref. mRNA	Protein	Cov.	CNV (fold)	AF	clinical significance
Lim1215	chr3:41266124	CTNNB1	c.121A>G	SNV	3	NM_001904.3	p.Thr41Ala	1997		100	path, lik path
	chr15:66774159	MAP2K1	c.635G>A	SNV	6	NM_002755.3	p.Ser212Asn	1997		100	prob path
Lim1215 R1	chr3:41266124	CTNNB1	c.121A>G	SNV	3	NM_001904.3	p.Thr41Ala	1739		100	path, lik path
	chr12:25398285	KRAS	c.34G>C	SNV	2	NM_033360.3	p.Gly12Arg	1607		49.41	path
	chr15:66774159	MAP2K1	c.635G>A	SNV	6	NM_002755.3	p.Ser212Asn	1997		100	prob path
Lim1215 R2	chr3:41266124	CTNNB1	c.121A>G	SNV	3	NM_001904.3	p.Thr41Ala	736		100	path, lik path
	chr12:25398281	KRAS	c.38G>A	SNV	2	NM_033360.3	p.Gly13Asp	980		50.51	path, lik path
	chr15:66774159	MAP2K1	c.635G>A	SNV	6	NM_002755.3	p.Ser212Asn	1346		100	prob path
DiFi	chr7:55198955	EGFR		CNV					10.7		path, lik path
DiFi R1	chr7:55198955	EGFR		CNV					6.7		path, lik path
	chr12:25364760	KRAS		CNV					21.7		path, lik path
DiFi R2	chr7:55198955	EGFR		CNV					6.7		path, lik path
	chr12:25364760	KRAS		CNV					28.7		path, lik path

Suppl. table 1: Next generation sequencing results (Oncomine Focus Assay)

AF, allele frequency; **cDNA**, complementary DNA; **CNV**, copy number variation; **Cov.**, Coverage; **lik path**, likely pathogenic; **path**, pathogenic; **prob path**, probably pathogenic (based on prediction algorithms, no information in ClinVar); **SNV**, single nucleotide variant

Lim1215 cetuximab treated

Rank	Gene names	log2 FC	"-log(p_adj)"
1	EPHA1	1,274564743	3,3110129
2	EPHA2	1,27055645	2,61315458
3	TGFBR1	1,235226075	1,1671055
4	MLKL	1,126009782	3,8482497
5	PIK3CA	1,073595524	2,06970671
6	POMK	0,956455072	4,56944673
7	MAPK6	0,888480028	1,07951107
8	CDK8	0,870285034	2,10149655
9	ATR	0,866937002	2,03912947
10	PBK	0,850308259	3,13839952
11	PRKCI	0,841203372	2,3702287
12	CAMK4	0,832149982	3,079231
13	PLK1	0,8037467	1,95116548
14	CDK9	0,801906745	2,47733245
15	MAPK14	0,801093419	3,13022096
16	MAP2K1	0,784678459	4,18940419
17	CSNK1G2	0,783434391	1,62862765
18	ADRBK1	0,773893356	3,35425415
19	EGFR	0,773827235	2,08404717
20	PRKCE	0,750103315	1,79324922
21	GRK6	0,746706645	2,65787189
22	ACVR1	0,728495121	2,48605498
23	FRK	0,695926666	1,90992439
24	PIK3R4	0,687929948	1,8015082
25	MET	0,680924892	2,24394443

Lim1215 untreated

Rank	Gene names	log2 FC	"-log(p_adj)"
1	EPHA2	1,14034303	2,02111845
2	SCYL1	0,61746661	2,26540524
3	PXK	0,55812836	3,57640291
4	EPHA1	0,52683512	0,91350351
5	MET	0,47411489	1,06912444
6	SRC	0,45997969	1,06965208
7	PRKCD	0,45149358	0,91692353
8	RIOK3	0,40691837	0,84853068
9	POMK	0,39618031	0,59838905
10	NEK3	0,36072556	0,8317588
11	CSNK1G2	0,34410779	0,63547629
12	MAPK6	0,34278123	0,61269525
13	CSNK1G3	0,33609327	0,97854093
14	LIMK1	0,33242003	0,98225864
15	LMTK2	0,31947851	0,76594156
16	TGFBR1	3,04E-01	2,71E-01
17	ACVR1	0,29629962	1,31586214
18	RIOK1	0,28255018	1,00319602
19	RPS6KB2	0,27856159	0,80687021
20	SMG1	0,27541987	0,87544519
21	MTOR	0,24564997	0,63225135
22	EIF2AK4	0,24293534	3,19677906
23	MAP3K11	0,23903131	1,0783843
24	CDK8	0,23718007	0,48266882
25	PHKG2	0,23655621	0,69643722

Suppl. table 2: Kinase overexpression in resistant Lim1215 and DiFi cell lines
Top 25 overexpressed kinases in CMAB resistant Lim1215 and DiFi cell lines compared to parental cells in the CMAB treated and untreated setting. Log2 FC represent fold change difference of both resistant cell lines compared to their parental counterpart. -log(p) represents FDR corrected p value of the change.

DiFi cetuximab treated

Rank	Gene names	log2 FC	"-log(p_adj)"
1	MASTL	1,65573835	4,35745082
2	ADRBK1	1,26054096	2,89787209
3	MELK	1,2105848	3,39680999
4	TTK	1,20751826	3,57960373
5	MLKL	0,97436523	3,12294611
6	CIT	0,9346935	3,59039003
7	AURKB	0,90800158	2,14207447
8	RIOK1	0,79893478	0,95874581
9	JAK1	0,79883989	2,26830918
10	CHEK1	0,77092346	3,1246356
11	EPHA2	0,76349004	1,56433743
12	PRKCI	0,71169949	2,40562509
13	CDK8	0,68355497	1,67985447
14	CSNK1D	0,653368	1,74949673
15	TNK2	0,6208237	3,85303334
16	STK4	0,5790952	3,99459178
17	VRK1	0,57884343	1,97574363
18	SGK223	0,57099501	4,03071201
19	ERBB2	0,55374527	2,44483608
20	PIK3R4	0,54120175	1,09731308
21	BUB1B	0,53669691	1,16171811
22	PEAK1	0,53280179	2,47140144
23	MAP2K1	0,53183683	2,33052985
24	MET	0,52941847	2,03221531
25	MAP2K4	0,52622143	3,5973355

DiFi untreated

Rank	Gene names	log2 FC	"-log(p_adj)"
1	MAP2K6	1,21732855	1,76328902
2	ERBB2	1,12556966	0,22586989
3	TBK1	0,90518665	1,54513335
4	CHEK2	0,79019499	0,82240179
5	CDK8	0,75954549	2,55830943
6	ABL2	0,67202473	0,98516021
7	STK16	0,65002473	1,27194746
8	TNNI3K	0,59784476	2,63057749
9	SGK223	0,59568628	2,50598984
10	SCYL2	0,56676006	3,84182578
11	SYK	0,55220652	0,41563186
12	SLK	0,55026515	1,74145271
13	LATS1	0,50908995	0,90723856
14	AKT1	0,47522688	1,15474163
15	PRKCA	0,46668657	0,7199697
16	MAP2K3	0,44967508	1,62101756
17	MAPKAPK2	0,4479332	1,58418001
18	MAP3K4	0,44114987	1,50517891
19	CIT	0,43852011	2,43168224
20	VRK2	0,42497476	1,32740823
21	PDK2	0,41370646	0,82562402
22	EPHA2	0,41300853	0,49137482
23	IGF1R	0,40715154	0,47432772
24	CSK	0,40080976	2,65411194
25	TGFBR2	0,38802497	0,74497334

Suppl. table 2: Kinase overexpression in resistant Lim1215 and DiFi cell lines
Top 25 overexpressed kinases in CMAB resistant Lim1215 and DiFi cell lines compared to parental cells in the CMAB treated and untreated setting. Log2 FC represent fold change difference of both resistant cell lines compared to their parental counterpart. -log(p) represents FDR corrected p value of the change.

Supplementary materials and methods

LC-MS/MS

Protein digestion, TMT-labeling and peptide fractionation

Cell lysates were adjusted to 1mg/ml and 100µg proteins from each biological replicate were reduced with 10mM DTT for 45min at 37°C and alkylated with 55mM chloro-acetamide for 30min at room temperature in the darkness. Lysates were diluted with 5 volumes of 40mM Tris-HCl, pH 7.6 to decrease the concentration of urea prior trypsinization. Digestion was performed by adding trypsin (Promega, Mannheim, Germany) in a 1:50 (w/w) enzyme-to-substrate ratio and incubating overnight at 37°C in a thermoshaker at 700rpm. On the next day samples were acidified by addition of formic acid (FA) to a concentration of 0.5% (v/v) to stop proteolysis and desalted using self-packed stage-tips (10 discs, Ø 1.5mm, C18 material, 3M EmporeTM Octadecyl C18, Saint Paul, MN, USA; wash solvent: 0.1% FA; elution solvent: 60% ACN in 0.1% FA). Peptide solutions were frozen at -80°C and dried in a SpeedVac. 100µg of protein digests were dissolved in 50mM HEPES, pH 8.5 and mixed for 10min at 20°C. TMT reagent (Thermo Fisher Scientific), dissolved in 100% anhydrous ACN was added to each vial to the final concentration of 11.6mM. Samples were incubated at 400rpm on a thermomixer for 1 hour at 20°C. The reaction was stopped by adding hydroxylamine to a final concentration of 0.4% and incubated for 15 min at 20°C at 400rpm. Labeled peptide solutions were pooled and desalted on 50mg Sep-Pak tC18, reversed-phase (RP) solid-phase extraction cartridges (Waters Corp., Finglas, Ireland; wash solvent: 0.1% FA; elution solvent: 60% CAN in 0.1% FA). TMT-labeled sample pools were fractionated on a Trinity P1 column (Thermo Fisher Scientific) using a Dionex Ultimate 3000 HPLC System (Dionex Corporation, Idstein, Germany) at a flow rate of 300µl/min. 100µg of protein digests were loaded onto the column in solvent A (10mM ammonium acetate in water, pH 4.7) and separated in a gradient of solvent B (95% acetonitrile in 10mM ammonium acetate, pH 5.4). Samples were collected in 32 fractions, dried down in a SpeedVac and measured on LC-MS.

LC-MS/MS analysis

1µg of each fraction was resuspended in 0.1% FA in water and injected into an Ultimate 3000 SD HPLC (Thermo Fisher Scientific) coupled to a Q Exactive HF (Thermo Fisher Scientific). Peptides were delivered to a trap column (75µm x 2cm, packed in house with 5µm C18 resin, Reprosil PUR AQ, Dr. Maisch, Ammerbuch, Germany) for 10 min at a flow rate of 5µl/min in loading solvent (0.1% FA in water) and separated on the analytical column (75µm x 45cm, packed in house with 3µm C18 resin, Reprosil Gold, Dr. Maisch) using a 90 min gradient (solvent A: 0.1% FA, 5% DMSO; solvent B: 0.1% FA, 5% DMSO in ACN). The Q-Exactive HF was operated in data-dependent acquisition (DDA) and positive ion mode, automatically switching between MS1 and MS2. Full-scan MS1 spectra were acquired from 360 to 1300m/z, 60,000 resolution, automatic gain control (AGC) target value of 3 x 106 charges and a maximum injection time (maxIT) of 10ms. We allowed up to 25 precursor ions to be selected for fragmentation. MS2 spectra were acquired at 100 to 1200m/z at 30,000 resolution to enable resolution of all TMT labels, AGC target value of 1 x 103 charges and maximum injection time of 55ms.

Database searching and data analysis

Peptide and protein identification and quantification were performed using MaxQuant (v1.5.5.1) with embedded Andromeda search engine. Spectra were searched against the UniProt databases (human, 48556 entries, downloaded on 19.07.2017). Trypsin/P was selected as proteolytic enzyme and up to two missed cleavage sites were allowed. Carbamido-methylation of cysteine was set up as fixed modifications and oxidation of methionine, and-N-terminal protein acetylation as variable modifications. The mass tolerance of precursor ions was set to 5ppm and for fragmentation to 20ppm. An FDR cut-off of 0.01 was used for identification of peptides and proteins. To facilitate further data analysis, the results were imported into the MaxQuant associated software suite Perseus (v.1.5.4.1). Quantification was performed TMT based. A Benjamini-Hochberg FDR corrected two-sided student's *t* test was used to assess statistical significance (FDR<0.05, S0 of 0.1).

Transcriptomic and clinical data

Patients and samples

Patient recruitment and sample acquisition for the Prospect-C trial have been described previously [1]. Of the 35 recruited *RAS* wildtype patients treated with single-agent cetuximab with successful baseline (BL) tissue sequencing, 15 patients showed prolonged treatment benefit as defined by RECIST criteria before progressive disease (PD). CET resistance was attributable to genomic alterations in the RAS/RAF pathway in 5/15 (33%) patients, as identified by tissue biopsy sequencing. Transcriptomic data was available for 3/5 patients (C1005, C1027, C1037).

Somatic mutation analysis, DNA copy number aberration and ctDNA-sequencing

Tissue biopsy exome sequencing and DNA copy number variation analysis were described elsewhere [1].

Transcriptomic data

Gene expression data from the Prospect-C trial were generated as described previously [1].

Materials

Primer sequences

Name	UPL	Sequence	Manufacturer
EPHA2 F	#89	CCCCAAGTTCGCTGACAT	biomers.net GmbH
EPHA2 R	#89	GGGGAGCCGGATAGACAC	biomers.net GmbH
GAPDH F	#60	AGCCACATCGCTCAGACAC	biomers.net GmbH
GAPDH R	#60	GCCCAATACGACCAAATCC	biomers.net GmbH
EFNA1 F	#43	GGAGAAGAGACTTGCAGCAGA	biomers.net GmbH
EFNA1 R	#43	TCCAGGCAAGTGGGAAGA	biomers.net GmbH

All primers were used in 500nM working concentrations; UPL – universal probe library, Hoffmann-La Roche

Antibodies

Name	Clone #	Manufacturer
α-EPHA2	1C11A12	Invitrogen
α-EPHA2	AF3035	R&D Systems
α-GFP	AF4240	R&D Systems
α-Tubulin	TU-01	Invitrogen

siRNA

Name	Target sequence	Manufacturer
siEphA2	CAGCGCCAAGUAAACAGGGUA	Qiagen
siControl xx	ACAACGAAGCUCCGUCCCUACCGAA	Thermo Fisher Scientific

Supplementary references

1. Woolston A, Khan K, Spain G, et al. Genomic and Transcriptomic Determinants of Therapy Resistance and Immune Landscape Evolution during Anti-EGFR Treatment in Colorectal Cancer. *Cancer Cell*. 2019;36(1):35-50.e9. doi:10.1016/j.ccell.2019.05.013
